# Optical Histology: A Method to Visualize Microvasculature in Thick Tissue Sections of Mouse Brain

**DOI:** 10.1371/journal.pone.0053753

**Published:** 2013-01-23

**Authors:** Austin J. Moy, Matthew P. Wiersma, Bernard Choi

**Affiliations:** 1 Beckman Laser Institute, University of California Irvine, Irvine, California, United States of America; 2 Department of Biomedical Engineering, University of California Irvine, Irvine, California, United States of America; 3 Edwards Lifesciences Center for Advanced Cardiovascular Technology, University of California Irvine, Irvine, California, United States of America; Tufts University, United States of America

## Abstract

**Background:**

The microvasculature is the network of blood vessels involved in delivering nutrients and gases necessary for tissue survival. Study of the microvasculature often involves immunohistological methods. While useful for visualizing microvasculature at the µm scale in specific regions of interest, immunohistology is not well suited to visualize the global microvascular architecture in an organ. Hence, use of immunohistology precludes visualization of the entire microvasculature of an organ, and thus impedes study of global changes in the microvasculature that occur in concert with changes in tissue due to various disease states. Therefore, there is a critical need for a simple, relatively rapid technique that will facilitate visualization of the microvascular network of an entire tissue.

**Methodology/Principal Findings:**

The systemic vasculature of a mouse is stained with the fluorescent lipophilic dye DiI using a method called “vessel painting”. The brain, or other organ of interest, is harvested and fixed in 4% paraformaldehyde. The organ is then sliced into 1 mm sections and optically cleared, or made transparent, using FocusClear, a proprietary optical clearing agent. After optical clearing, the DiI-labeled tissue microvasculature is imaged using confocal fluorescence microscopy and adjacent image stacks tiled together to produce a depth-encoded map of the microvasculature in the tissue slice. We demonstrated that the use of optical clearing enhances both the tissue imaging depth and the estimate of the vascular density. Using our “optical histology” technique, we visualized microvasculature in the mouse brain to a depth of 850 µm.

**Conclusions/Significance:**

Presented here are maps of the microvasculature in 1 mm thick slices of mouse brain. Using combined optical clearing and optical imaging techniques, we devised a methodology to enhance the visualization of the microvasculature in thick tissues. We believe this technique could potentially be used to generate a three-dimensional map of the microvasculature in an entire organ.

## Introduction

The survival of mammalian tissue is dependent on the delivery of nutrients and growth factors and the exchange of oxygen and carbon dioxide gases. Nutrient delivery and gas exchange is done through the blood, which travels through the large feeder blood vessels of the vascular system to a network of small blood vessels and finally to the tissue. This network of small blood vessels is known as the microvasculature and its primary functions are twofold: 1) to transport blood to the tissue 2) in doing so, to facilitate delivery of essential nutrients from the blood and exchange gases with the surrounding tissue that are necessary for tissue survival. Thus, the microvasculature plays a pivotal role in the survival and function of tissue and, as a result, the study of the microvasculature in both normal and diseased states is important. The role of the microvasculature has previously been investigated in tumors [1]–[4], the cardiovascular system [5]–[7], the brain [8], the eye [9], [10], and skin [11], [12].

The gold-standard method of visualizing tissue microvasculature is the well-established immunohistochemistry technique [13]–[15]. Briefly, immunohistochemistry involves the staining of thin tissue slices with fluorescently-labeled antibodies that bind selectively to molecular markers of interest in the tissue, such as the CD31 marker for microvasculature. Once the tissues are stained, a fluorescence microscope is used to visualize and obtain images of the markers that can aid researchers in investigating and understanding the disease state of the tissue. The tissue slices used in immunohistochemistry are typically 1–5 µm in thickness and allow for very detailed, cellular-level interrogation of tissue. Using immunohistochemistry to visualize microvasculature in tissue yields extremely detailed images, but is limited to small spatial regions of interest, typically on the order of hundreds of µm. A single immunohistology slice will allow visualization of several microvessel cross-sections, but not an entire microvessel, let alone a network of microvessels.

While interrogation of tissue microvasculature at the cellular level is important, interrogation of the microvasculature at the organ level can elucidate the global changes in the microvasculature that affect and also are affected by tissue disease state. Immunohistochemistry is well-suited for the study of small µm-size spatial regions, but is impractical to study the microvasculature of an entire organ, which is on the order of cm in size.

An alternative technique to enhance visualization of the microvasculature is “vessel painting” [16]–[18]. First described in 2002 [16], the technique was originally used to enhance visualization of the microvasculature in the retina. A more detailed protocol published in 2009 [18] describes methodology to collect detailed images of the microvasculature in other organs such as heart, skin, and brain. The technique involves cardiac perfusion of a contrast agent, typically a fluorescent dye, into the systemic vasculature, to label the blood vessels. A fixative is then perfused to achieve a fluorescent “cast” of the microvasculature. The tissue of interest is harvested and incubated in the fixative, after which it is imaged typically with confocal fluorescence microscopy.

**Figure 1:**
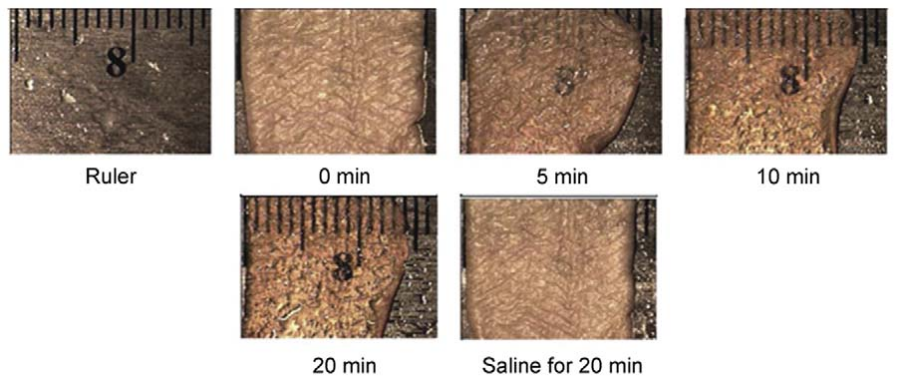
Time-lapse image demonstrating optical clearing effect on skin. The sample was immersed in 14M glycerol and placed on top of a ruler at specific time points and imaged with a digital camera. As the tissue becomes more transparent, the underlying ruler becomes easier to see. The effect is reversible with saline incubation.

While this vessel painting technique can yield highly detailed images of the microvasculature, these images are limited to the superficial imaging depth typically associated with optical microscopes. The depth is restricted due to the scattering nature of most biological tissues to optical wavelengths [19]. To overcome optical scattering, a technique known as optical clearing can be used [20], [21]. Optical clearing was used in previous studies to enhance visualization of microvasculature in breast tumors [22], brain [23], pancreas [24], and in spinal cord tissue [25]. Briefly, optical clearing involves the use of a class of hyperosmotic chemical agents [26], [27] that, when incubated with tissue, reduce the optical scattering in the tissue [28], [29] and increase the optical transparency of the treated tissue (Figure 1). With a reduction in tissue scattering, fluorescence excitation can penetrate more deeply and increased fluorescence emission can propagate to the surface from deeper depths.

Previously obtained data demonstrate that the combination of optical clearing and vessel painting can provide detailed images of the microvasculature [24], [30], [31]. In these studies, mouse microvasculature was labeled with an intracardiac injection of DiI, a lipophilic carbocyanine dye that embeds itself in the lipid membrane of cells. In one of these studies, the small intestine was harvested and then rendered more transparent with a chemical agent called FocusClear (CelExplorer Labs, Taiwan). The fluorescently-labeled microvasculature in the optically cleared tissue was imaged using confocal fluorescence microscopy. While the images of the microvasculature were quite detailed, the imaging field of view was limited both in depth (∼300–400 µm) and to a small field of view.

To address the limited imaging depth, we present here a technique that facilitates imaging of the entire microvascular network of an entire organ. We developed an experimental protocol, which we have dubbed “optical histology,” that utilizes vessel painting, optical clearing of the tissue, and optical imaging to enhance the visualization of tissue microvasculature.

## Materials and Methods

### Labeling of Microvasculature

Mouse brains were acquired from 2–3 month old C3H male mice with body mass of 25–30 g. The microvasculature is labeled *in vivo* via the vessel painting method described previously [17], [18]. Briefly, the carbocyanine fluorescent dye 1,1′-dioctadecyl-3,3,3′3′-tetramethylindocarbocyanine perchlorate (DiI) (Invitrogen, Carlsbad, CA), a lipophilic dye that embeds in the lipid membranes of the endothelial cells lining the walls of the microvasculature, was perfused via cardiac perfusion to label the microvasculature. A solution of 4% w/v paraformaldehyde was then perfused intracardially. Afterwards, tissues were excised and further fixed in 4% w/v paraformaldehyde for a minimum of 72 hours.

### Tissue slicing

**Figure 2:**
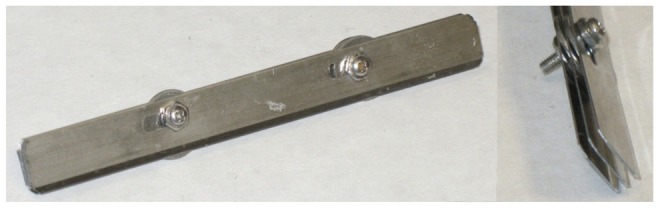
Multi-section scoring device, consisting of histology blades connected together, used to score the tissue embedded in agarose gel. Individual tissue slices are then made using a single histology blade.

Post-fixation, mouse brains were embedded in 5% agarose gel and then scored into several 1 mm thick coronal sections using a custom-built multi-section scoring device (Figure 2) that consisted of several connected microtome blades. This results in an agarose-embedded brain that has lines cut into the agarose gel to demarcate each 1 mm section. Using these lines, coronal slices were then sliced using individual microtome blades (Leica Low-profile Disposable Blades, Leica Microsystems GmbH) into the 1 mm thick slices and excess agarose trimmed from the tissue.

### Optical clearing

**Figure 3:**
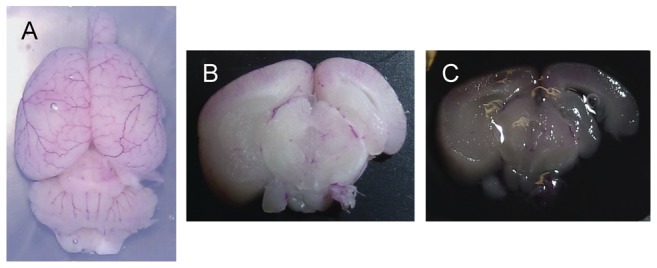
Progression from DiI-stained brain to optically-cleared coronal brain slice. (A) DiI-stained brain embedded in agarose gel. (B) Uncleared coronal brain slice. (C) Same slice in (B) after immersion in optical clearing agent.

Tissue slices were incubated in 0.3 ml of FocusClear (CelExplorer Labs, Hsinchu, Taiwan), a DMSO-based optical clearing agent, for three hours (Figure 3). After optical clearing, the tissues were then mounted directly between two 50×22 mm size cover glasses (Fisherfinest Premium Cover Glass, Fisher Scientific) in preparation for confocal microscopy. Two cover glasses are necessary to sandwich the tissue sample to minimize curling of the tissue onto itself as a result of incubation in FocusClear.

### Confocal fluorescence microscopy

A Zeiss LSM 510 Meta (Carl Zeiss AG, Oberkochen, Germany) laser scanning confocal microscope was used for tissue imaging. The DiI dye was excited with a 543 nm laser source and fluorescence emission collected with a 565–615 nm bandpass filter in front of the detector. Image z-stacks were collected with a 10× objective (Plan-Neofluar 10×/0.30 Ph1), giving a system lateral resolution of 0.7 µm and axial resolution of 9 µm. Image dimensions were 900 µm×900 µm with z-slice interval of 14 µm. Sequential image z-stacks were then acquired, resulting in a mosaic of image z-stacks that spanned the entire tissue slice. To evaluate the overall technique, microvessels were visualized in 20 optically cleared brain slices and a representative sample of this data is presented here.

### Image Processing

**Figure 4:**
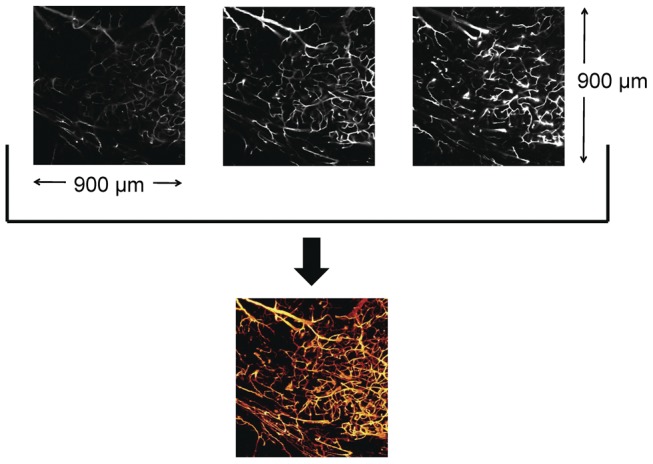
Protocol used to generate maximum intensity projection (MIP) images, which are a conglomeration of the brightest pixels from all images in a three-dimensional stack. (Top) Representative z-stack confocal fluorescence images of the microvasculature. (Bottom) MIP image, with each color in the false colormap corresponding to a specific depth in the stack.

Image z-stacks were processed using custom processing code written in MATLAB (Mathworks, Natick, MA). In addition, the open source MATLAB functions tiffread29.m [32], show_stack.m, and show_image.m, which collectively facilitate manipulation of image z-stack data acquired using a Zeiss microscope and saved in. LSM format, were integrated into the custom MATLAB code. The images were processed as both maximum intensity projection (MIP) images and 3D fly-through movies. A MIP image consists of the maximum intensity pixel found in the z-stack at each pixel location. MIP images were calculated from each image z-stack (Figure 4) and then tiled together to produce a MIP image of the entire tissue slice. Each pixel in the MIP was then assigned a color specifying its depth in the z-stack, resulting in a depth-encoded MIP of the entire tissue slice. The calculated MIP acts as a map of the vasculature and was then used to calculate the functional vascular density (FVD) for specific regions of interest using a previously written algorithm [33]. Briefly, FVD was calculated by first thresholding imaged vessels via size and pixel intensity, followed by skeletonization over the entire image. The total length of vessels in a given tissue region of interest is then automatically computed by the algorithm and divided by the region of interest area to calculate the FVD. FVD was calculated in two distinct regions of interest (ROIs) in each of five separate areas of each brain slice, totaling 10 ROIs in each brain slice. The average size of each ROI was 0.011 cm^2^, ranging from 0.034 cm^2^ to 0.004 cm^2^. Three-dimensional fly-through movies in the coronal plane of the tissue microvasculature were made by tiling together image data from each slice in the image z-stack, effectively resulting in an image z-stack for the entire tissue slice. Using ImageJ, the image z-stack of the entire tissue slice was converted into a movie file for subsequent playback (Video S1).

## Results

**Figure 5:**
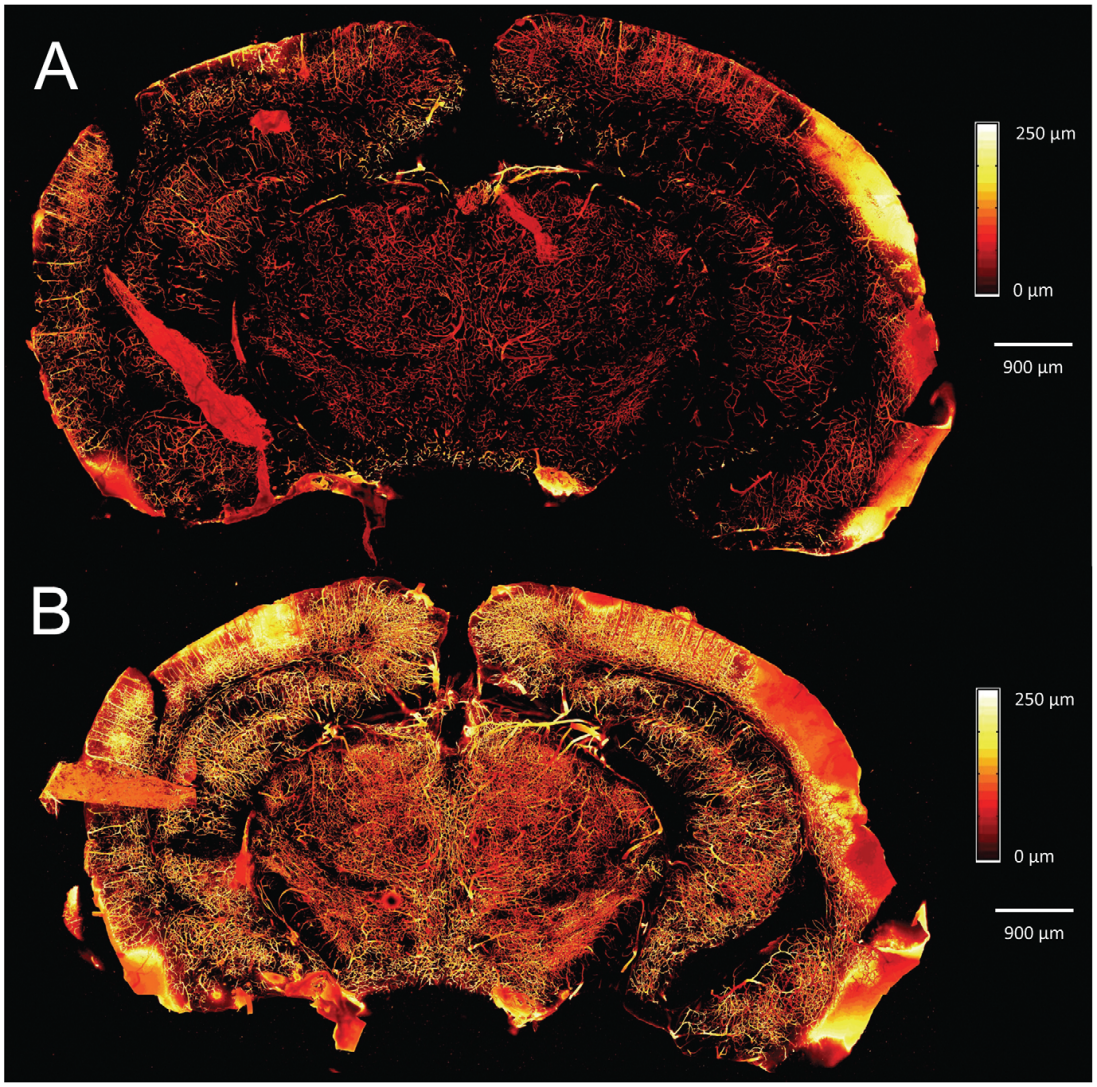
Depth-encoded MIP images of brain slice microvasculature (a) before and (b) after optical clearing with FocusClear. Both images are over the same interrogation volume (250 µm) to show the increase in lateral microvasculature visualized due to optical clearing.

**Figure 6:**
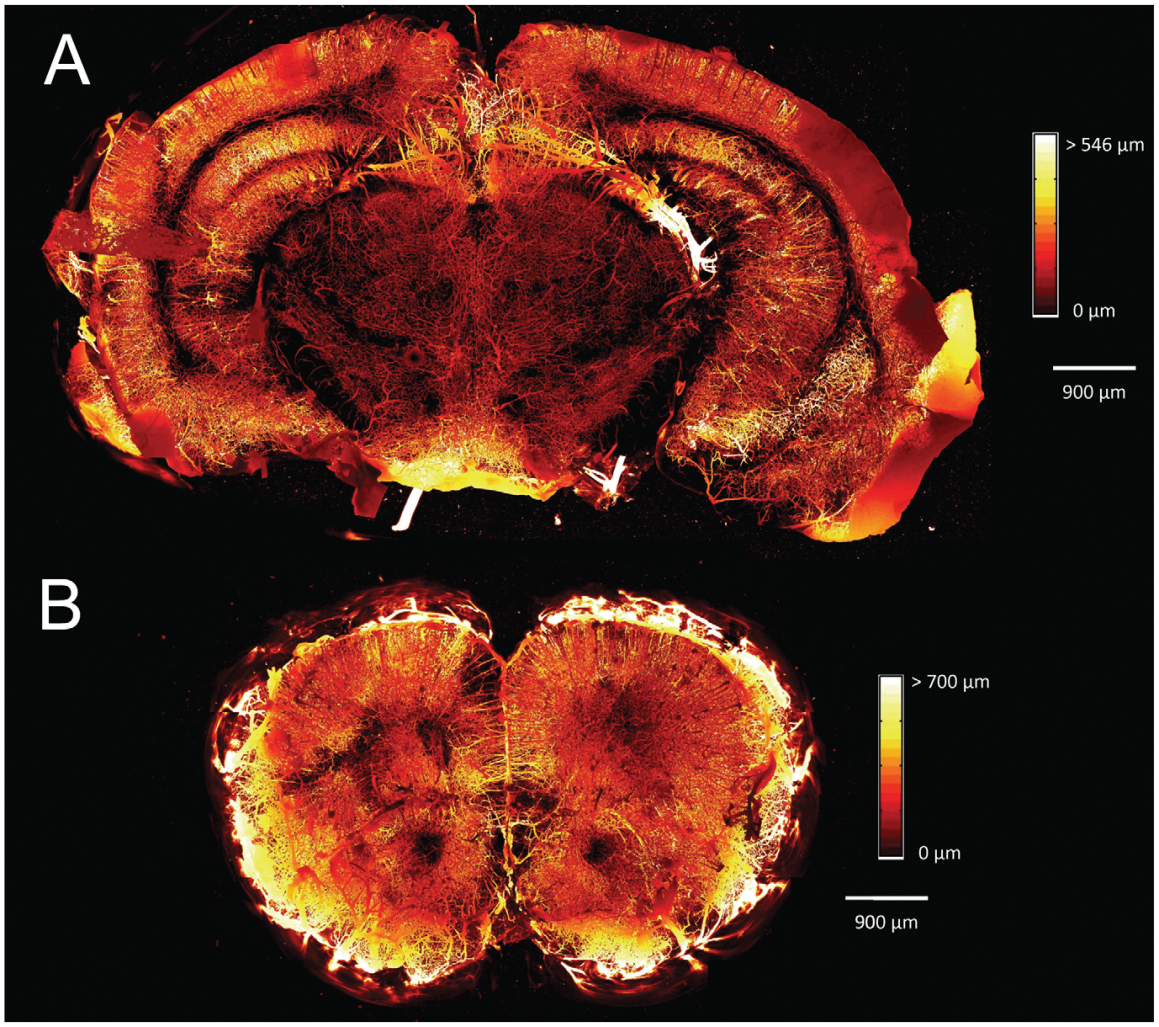
Representative examples of Focus Clear-enhanced visualization of the cerebral microvasculature. (a) Depth-encoded MIP image of the brain slice in Figure 5 over the entire interrogation volume of 850 µm (b) Depth-encoded MIP image of a similar brain slice optically cleared with FocusClear from a different brain. Microvasculature is visualized to a depth of 820 µm in this slice.

**Figure 7:**
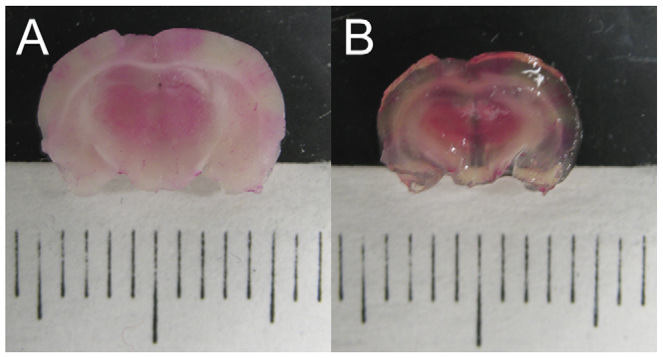
Images showing effect of FocusClear on tissue size. The brain slice dimensions were reduced approximately 20% after 3 hours of incubation in FocusClear.

Figure 5 is a set of depth-encoded, MIP images for a single coronal brain slice approximately 4 mm into the brain at bregma. Figure 5A is the native slice before optical clearing, showing microvasculature up to a depth of 250 µm. Figure 5B is a MIP image of the same slice and to the same 250 µm depth as shown in Figure 5A, after optical clearing. The effect of using optical clearing to enhance visualization of the microvasculature, in particular the lateral microvascular density, is clearly shown. Figure 6A shows a depth-encoded MIP for the same brain slice shown in Figure 5, but over the increased tissue depth (850 µm) that is accessible after optical clearing. Figure 6B shows a depth-encoded MIP image of an optically-cleared coronal brain slice from a different brain, which also was located approximately 4–5 mm into the brain (approximately at bregma). The FVD was calculated in several regions of interest in different areas of the MIP images in Figures 5 and 6 and the values are summarized in Table 1. Figure 7 is a visual comparison of a coronal brain slice before and after it was optically cleared. After clearing, the brain slice was reduced in size by ∼20%. The video in Video S1 shows a representative three-dimensional “fly-through” movie generated from the data shown as a MIP image in Figure 6B and demonstrates that the microvasculature in the brain slice exhibits local changes at different imaging depths.

**Table 1:** Table summarizing FVD calculated from MIP maps of the microvasculature in Figures 5 and 6. FVD was averaged in two different regions of interest in five separate areas of the brain (top right, top left, bottom right, bottom left, and middle).

Brain slice	Tissue volume depth	Functional Vascular Density (ROI)
Figure 5A	250 µm	444 cm^-1^ (top right)
		296 cm-1 (bot right)
		296 cm^-1^ (bot left)
		361 cm^-1^ (top left)
		252 cm^-1^ (middle)
Figure 5B	250 µm	826 cm^-1^ (top right)
		635 cm-1 (bot right)
		514 cm^-1^ (bot left)
		655 cm^-1^ (top left)
		633 cm^-1^ (middle)
Figure 6A	850 µm	793 cm^-1^ (top right)
		799 cm-1 (bot right)
		891 cm^-1^ (bot left)
		868 cm^-1^ (top left)
		611 cm^-1^ (middle)
Figure 6B	820 µm	928 cm^-1^ (top right)
		908 cm-1 (bot right)
		806 cm^-1^ (bot left)
		888 cm^-1^ (top left)
		822 cm^-1^ (middle)

## Discussion

The results demonstrate our ability to visualize the tissue microvasculature with encoded depth information. Using our combined optical clearing and optical imaging technique, we were able to create an extremely detailed map of the microvasculature in coronal sections of mouse brain. We were also able to resolve 5 µm diameter capillaries in the tissue, even at a relatively low 10× magnification, which demonstrates the advantages of using DiI in the cardiac perfusion and the enhanced fluorescence signal due to optical clearing of the tissue. Additionally, the tissue imaging depth was limited by both the thickness of the cover slip used and the working distance (5.2 mm) of the 10× objective.

To assess the performance of our technique, we directly compared MIP images of a brain slice before and after optical clearing with FocusClear and calculated the FVD for both conditions. The brain slice in Figure 5 was imaged, rehydrated, immersed in FocusClear for 3 hours, and then imaged again. We first calculated the FVD in several regions of interest in different areas of the native slice (Figure 5A) and after clearing (Figure 5B) over the same interrogation volumes. This analysis enabled quantitative assessment of the degree to which optical clearing enhanced the lateral visualization of the microvasculature. Comparing the FVD values in Table 1 for Figure 5A and Figure 5B, the FVD of the slice over the same interrogation volume increased by up to a factor of two.

We then assessed how the increased penetration depth afforded by optical clearing affected the FVD by calculating the FVD in several regions of interest of the MIP image over the entire interrogation volume in this same brain slice (Figure 6A). Similar to Figure 5B, the MIP in Figure 6A demonstrated an increase in lateral microvasculature as compared to the native slice in Figure 5A, and was reflected in the calculated FVD values. In addition, optical clearing enabled a ∼240% increase in the depth over which the vasculature could be visualized (Figures 5A and 6). Together, the increase in lateral microvasculature and increase in tissue depth resulted in an increase in FVD by about 2–3×. Because optical clearing of the tissue enables visualization deeper in the tissue, and hence a greater tissue interrogation volume, it is expected that the FVD would be larger since more of the deeper vessels in the tissue can be visualized. This is verified by comparing the MIP in Figure 6A to the MIP in Figure 5B, which is the same optically cleared brain slice but created over different interrogation volumes. The FVD in Figure 6A is larger by about 1.5× due to the additional volume. This demonstrates how optical clearing of the tissue allows for a more accurate determination of the FVD by allowing enhanced visualization of microvessels both laterally and several hundred µm into the tissue.

To assess the repeatability of our technique, we looked at another optically cleared brain slice (Figure 6B) from a completely different brain. This brain slice clearly showed a considerable increase in observed microvasculature compared to the native slice in Figure 5A. To quantify this, we calculated FVD in this slice in several regions of interest and overall, the FVD increased by about 2–3× compared to the native slice in Figure 5A. The FVD values were also very similar to those calculated in Figure 6A, showing that optical clearing of similar brain slices in two different brains yields similar FVD values.

The three-dimensional fly-through movie (Video S1) gives an intuitive and detailed view of the tissue microvessel network as it changes at each depth of the tissue slice. This movie demonstrates the ability of the optical histology technique to allow detailed visualization of tissue microvasculature in thick tissue. Additionally, it serves as a precursor to a three-dimensional map of the microvasculature in an entire organ, which could be achieved by putting together three-dimensional fly-through tissue microvasculature movies of several sequential tissue slices. This three-dimensional map of the microvasculature would be very significant in furthering the study of the microvasculature by giving structural and functional information about the microvasculature at the organ level.

Tissue microvasculature has previously been imaged to a depth of 2 mm using optical frequency domain imaging (OFDI) [34]. While the microvasculature can be imaged to a significant depth using OFDI, this imaging modality is meant more for *in vivo* investigation and would be less appropriate for creating a three-dimensional map of the microvasculature in an intact thick tissue or organ. The key advantage of optical histology is in enhancing the ability of confocal microscopy and other optical methods such as nonlinear optical microscopy. These methods excel at producing highly detailed images of the microvasculature in isolated areas at superficial depths. With optical histology, we can expand the scope of these imaging methods to produce highly detailed images of the microvasculature several hundreds of µm into the tissue over wide fields of view.

Enhanced tissue imaging with optical clearing is a concept that has been studied by several groups and has attracted significant attention [20]. A recent study [35] reports on a novel optical clearing agent (Sca*l*eA2) with a clearing effect similar to that of FocusClear. The proposed benefits of this optical clearing agent are low cost and the ability to formulate it with off-the-shelf reagents, compared to the proprietary nature of FocusClear. With Sca*l*eA2, the optimal optical clearing time for a 1 mm thick tissue was ∼48 hours. In contrast, with our method, the cerebral microvasculature maps were achieved with only a three-hour incubation time. Additionally, the brain slices cleared with Sca*l*eA2 became physically larger as they cleared [35]. In contrast, slices cleared with FocusClear experience a ∼20% reduction in size (Figure 7).

There is considerable interest in methods to produce physiological maps at the organ level. A recent study described the development of an automated micro-optical sectioning tomography instrument [36] to generate a map of brain physiology at the organ level. In this study, whole mouse brains were prepared for immunohistochemistry, and the entire process of slicing coronal brain slices and simultaneous imaging of the slices on a light microscope was automated. The group then took the stacks of image slices and calculated MIP images of the slices, resulting in high resolution images of the neural structures in the brain.

The time required to produce these images, however, is quite long. The tissue preparation required six months to allow sufficient time for the stain to diffuse throughout the entire brain. The image acquisition required 242 hours of continuous imaging. The time required for image processing, which is not explicitly stated in the publication, is reported to be “much longer than the acquisition time”. In contrast, the time and effort required with our protocol to produce a detailed map of the microcirculation at the organ level is substantially less. The key step in our protocol that enables a dramatic reduction in tissue preparation time is the slicing of the tissue into 1 mm thick sections. Although we report only on imaging of the microvasculature, we anticipate that other tissue structures and functional processes can be captured with use of the appropriate endogenous and/or exogenous fluorescent reporter. Table 2 summarizes the advantages of both techniques.

**Table 2:** Comparison of experimental parameters between optical histology and the automated micro-optical sectioning tomography (MOST) technique [36].

	MOST	Optical Histology
**Detail**	Microvasculature, neural structures	Microvasculature
**Acquisition time**	242 hours	24–30 hours
**Number of slices**	15,380	10
**Total time**	7 months	7 days

We have presented here a new technique called optical histology that utilizes combined optical clearing and optical imaging to acquire organ-level maps of the microvasculature in the brain. Previous work has presented maps of the microvasculature for small regions of interest, typically in the 100 s of µm. We have presented maps of tissue microvasculature over a 1 cm×1 cm spatial area by creating a mosaic of several adjacent image stacks, ultimately covering the entire tissue area. We have shown maps of the microvasculature as depth-encoded MIP images and as a three-dimensional fly-through movie, both of which give important information in an easily understood format. These images do not require expensive image rendering software to produce, instead calculated using a simple custom processing code written in MATLAB. This technique is a quick and efficient method to enhance the visualization of tissue microvasculature in three dimensions. Future work includes using this technique on several tissue slices to produce three-dimensional maps of the microvasculature in the entire brain, as well as applying this technique to visualizing microvasculature in other tissue types, such as skin, tumor, and heart models.

## Supporting Information

Video S1 Movie showing tiled confocal fluorescence images at different depths within the optically cleared coronal slice shown in Figure 6B. The movie reaches a final depth of 820 µm into the tissue.
(ZIP)
